# Transient pharmacologic lowering of Aβ production prior to deposition results in sustained reduction of amyloid plaque pathology

**DOI:** 10.1186/1750-1326-7-39

**Published:** 2012-08-14

**Authors:** Pritam Das, Christophe Verbeeck, Lisa Minter, Paramita Chakrabarty, Kevin Felsenstein, Thomas Kukar, Ghulam Maharvi, Abdul Fauq, Barbara A Osborne, Todd E Golde

**Affiliations:** 1 Department of Neuroscience, Mayo Clinic College of Medicine, 4500 San Pablo Rd S, Jacksonville, FL, 32224, USA; 2 Department of Veterinary & Animal Sciences, University of Massachusetts, 661 N. Pleasant St., Amherst, MA, 01003, USA; 3 Center for Translational Research in Neurodegenerative Disease, Department of Neuroscience, McKnight Brain Institute, College of Medicine, University of Florida, 1600 SW Archer Road, Gainesville, FL, 32608, USA; 4 Department of Pharmacology and Neurology, Emory University School of Medicine, 1510 Clifton Rd NE, 5123 Rollins Research Center, Atlanta, GA, 30322, USA

**Keywords:** Aβ, Alzheimer’s disease, APP, Therapeutics, γ-secretase inhibition

## Abstract

**Background:**

Alzheimer’s disease (AD) is the leading cause of dementia among the elderly. Disease modifying therapies targeting Aβ that are in development have been proposed to be more effective if treatment was initiated prior to significant accumulation of Aβ in the brain, but optimal timing of treatment initiation has not been clearly established in the clinic. We compared the efficacy of transient pharmacologic reduction of brain Aβ with a γ-secretase inhibitor (GSI ) for 1–3 months (M) treatment windows in APP Tg2576 mice and subsequent aging of the mice to either 15M or 18M.

**Results:**

These data show that reducing Aβ production in a 2-3M windows both initiated and discontinued before detectable Aβ deposition has the most significant impact on Aβ loads up to 11M after treatment discontinuation. In contrast, initiation of treatment for 3M windows from 7-10M or 12-15M shows progressively decreasing efficacy.

**Conclusions:**

These data have major implications for clinical testing of therapeutics aimed at lowering Aβ production, indicating that; i) these therapies may have little efficacy unless tested as prophylactics or in the earliest preclinical stage of AD where there is no or minimal Aβ accumulation and ii) lowering Aβ production transiently during a critical pre-deposition window potentially provides long-lasting efficacy after discontinuation of the treatment.

## Background

Alzheimer’s disease (AD) is the leading cause of dementia among the elderly. Numerous studies in humans have demonstrated the sequential development of various pathological features that characterize AD and the relationship of these pathologies to diagnosis of dementia [1]. These studies support the amyloid cascade hypothesis which posits that accumulation of Aβ aggregates triggers a series of downstream pathologies that results in clinical AD [2]. AD has recently been proposed to have three pre-clinical stages, characterized in sequence by the development of Aβ pathology (stage 1), the presence of plaque pathology, neurofibrillary pathology and initial evidence for neurodegeneration (stage 2), and finally by the presence of stage 2, pathologies plus evidence for subtle cognitive decline (stage 3). This progression likely occurs over a 10–20 year timeline [3].

The amyloid cascade hypothesis predicts that attenuating or preventing Aβ aggregation and accumulation will attenuate or prevent the subsequent development of AD. Strong experimental support for the amyloid hypothesis has supported the rationale for many therapeutic approaches targeting Aβ. Major investments have been made to develop inhibitors or modulators of Aβ production [4]. γ-secretase inhibitors (GSIs), β-secretase inhibitors (BSIs) and γ-secretase modulators (GSMs) have been or are currently being tested in human AD trials. Both the GSM tarenflurbil (R-flurbiporfren), and the GSI semagacestat, failed to show efficacy in phase III human trials [4,5]. Several other GSMs, GSIs, and BSIs are currently in early stage trials. Based on the clinical failures of tarenflurbil and semagacestat as well as other anti-Aβ therapies not targeting Aβ production but its aggregation or clearance, we and others have begun to question whether targeting of Aβ in symptomatic AD may be a futile endeavor [6].

**Figure 1:**
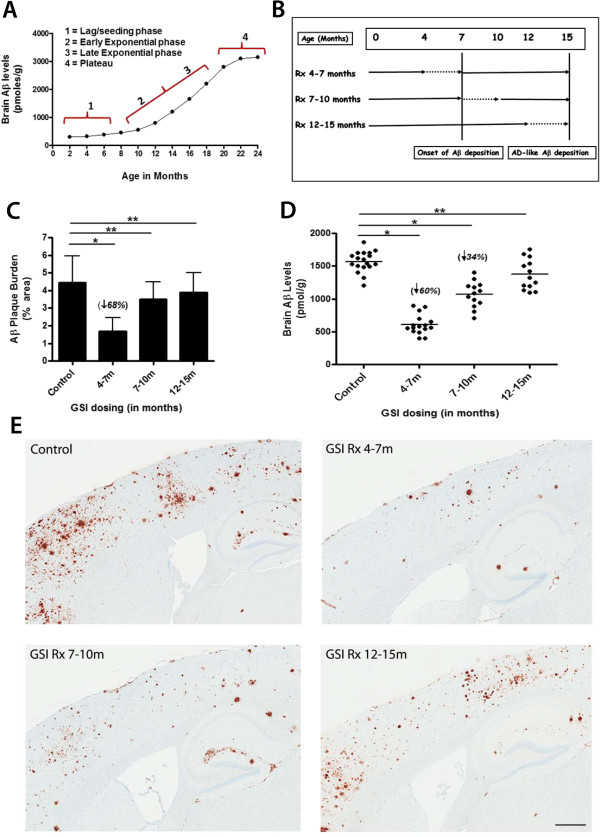
**Effects of LY window therapy on amyloid deposition. A**. Time course of Aβ accumulation in Tg2576 mice. FA solubilized brain Aβ42 + Aβ40 levels measured by ELISA are shown (*n* = 3–5 mice/age group)**. B**. Schematic of the various transient LY dosing strategies***.*** Dotted lines represent the transient LY treatment time points. **C**. Aβ plaque burden analysis of treated cohorts compared to controls (*p < 0.05, ** ns, ANOVA). **D**. Biochemical analyses (FA solubilized Aβ42 + Aβ40 levels) by ELISA showing Aβ levels. (*p < 0.05, **ns, ANOVA). **E**. Representative brain sections stained with anti-Aβ mAb showing Aβ plaques at 15M of age in the LY treated cohorts. Scale bar = 100 μm.

Given the framework provided by the amyloid cascade hypothesis, disappointing results from completed trials of anti-Aβ therapies in patients with AD, and several but limited preclinical studies supporting the concept that when targeting Aβ “earlier is better” [7-9], we explored the relationship between long-term efficacy with respect to reducing amyloid loads and timing and duration of Aβ lowering. In designing these studies, we were struck by the parallels between Aβ accumulation in the mouse brain and *in vitro* studies of Aβ aggregation. *In vitro*, Aβ aggregates in a nucleation dependent polymerization reaction, which exhibits three phases: a lag phase in which nucleation occurs but not fibril formation, an exponential growth phase in which fibril formation and growth occur rapidly, and a plateau phase where fibril growth and formation slows [10]. Except for being more extended in terms of time, studies in APP Tg2576 transgenic mice show that Aβ aggregation and accumulation follows a similar course in the brain [11]. There is an initial lag phase where no Aβ aggregation and accumulation occurs, followed by an exponential accumulation phase, and finally when amyloid loads become AD-like, the growth slows or plateaus (see Figure 1A). Given these parallels, we tested the efficacy of transient lowering of Aβ production in 1-3M treatment “windows” corresponding to these various phases of deposition using the GSI LY-411,575 (LY) in APP Tg2576 mice. These data show that GSI treatment during the pre-deposition window (4-7M) before the early exponential phase of deposition (7-10M) or late exponential phase (12-15M) has by far the most efficacy in terms of reduction of plaque loads at 15M of age.

## Results and discussion

### *In vitro and in vivo* dosing studies with Ly-411,575

Ly-411,575 is potent orally bioavailable γ-secretase inhibitor [12]. We synthesized large quantities for these studies and then performed both *in vitro* and *in vivo* studies to validate Ly-411,575 potency. The IC50 in cell culture for LY-411,575 was between 1–3 nm (see Additional file 1: Figure S1A). Six hours after a single IP dose, Aβ40 levels are reduced by ~75% in the brain of non-depositing Tg2576 mice (Additional file 1: Figure S1B). Oral administration (10 mg/kg/day) administered as a single dose reduces brain Aβ40 levels 24 hours after the final dose (7 days) by ~65% (Additional file 1: Figure S1C) and plasma Aβ40 by ~95% (Additional file 1: Figure S1D). Plasma Aβ levels are reduced more quickly than brain levels following oral dosing with maximal inhibition seen after 3 days (Additional file 1: Figure S1D). For long term *in vivo* mouse studies, we tested administration of Ly-411,575 using various dosing strategies and time points (Table 1). Oral administration (formulated in rodent chow) continually for 2 weeks with either - 1 mg/kg/day or 2.5 5 mg/kg/day reduced brain Aβ40 levels by ~50% and plasma Aβ40 by ~80%, without overt toxicities ( Table 1). However, continuous oral dosing (>2 weeks) using the higher dose of 5 mg/kg/day or 10 mg/kg/day resulted in overt toxicities in mice (Table 1). Therefore, in all long term studies presented in this manuscript, we have used the 2.5 mg/kg/day dose formulated in rodent chow.

**Table 1:** **Brain and plasma Aβ40 levels following *in vivo *GSI treatment in Tg2576 mice**

**Compound**	**Strain of Mice**	**Route**	**Dose**	**Length of Treatment**	**% Aβ40 Reduction*Brain***	**% Aβ40 Reduction*Plasma***	**Overt Toxicity**
LY-411, 575	Tg2576	Intraperitoneal	5 mg/kg	6 hrs	75%	92%	No
			10 mg/kg	6 hrs	77%	96%	No
			25 mg/kg	6 hrs	78%	92%	No
LY-411, 575	Tg2576	Oral (Suspension in Kool-Aid)	10 mg/kg/day	1 Day	47%	70%	No
				3 Days	65%	86%	No
				7 days	75%	95%	No
LY-411, 575	Tg2576	Oral (Formulated In Rodent Chow)	1 mg/kg/day	2 weeks	36%	67%	No
			2.5 mg/kg/day	2 weeks	51%	78%	No
			5 mg/kg/day	2 weeks	78%	91%	Yes*
			10 mg/kg/day	2 weeks	83%	95%	Yes*

Tg2576 mice (3 months old) were dosed with GSI LY-411,575 using various routes, time points and indicated doses. After GSI treatment, groups of mice (*n* =3/group) were sacrificed and mouse brains were harvested and extracted in 2% SDS. Brain and plasma Aβ40 levels were then measured by ELISA.

*Diarrhea and hair loss.

### Transient Aβ reduction dramatically reduces the subsequent accumulation of amyloid plaques

**Figure 2:**
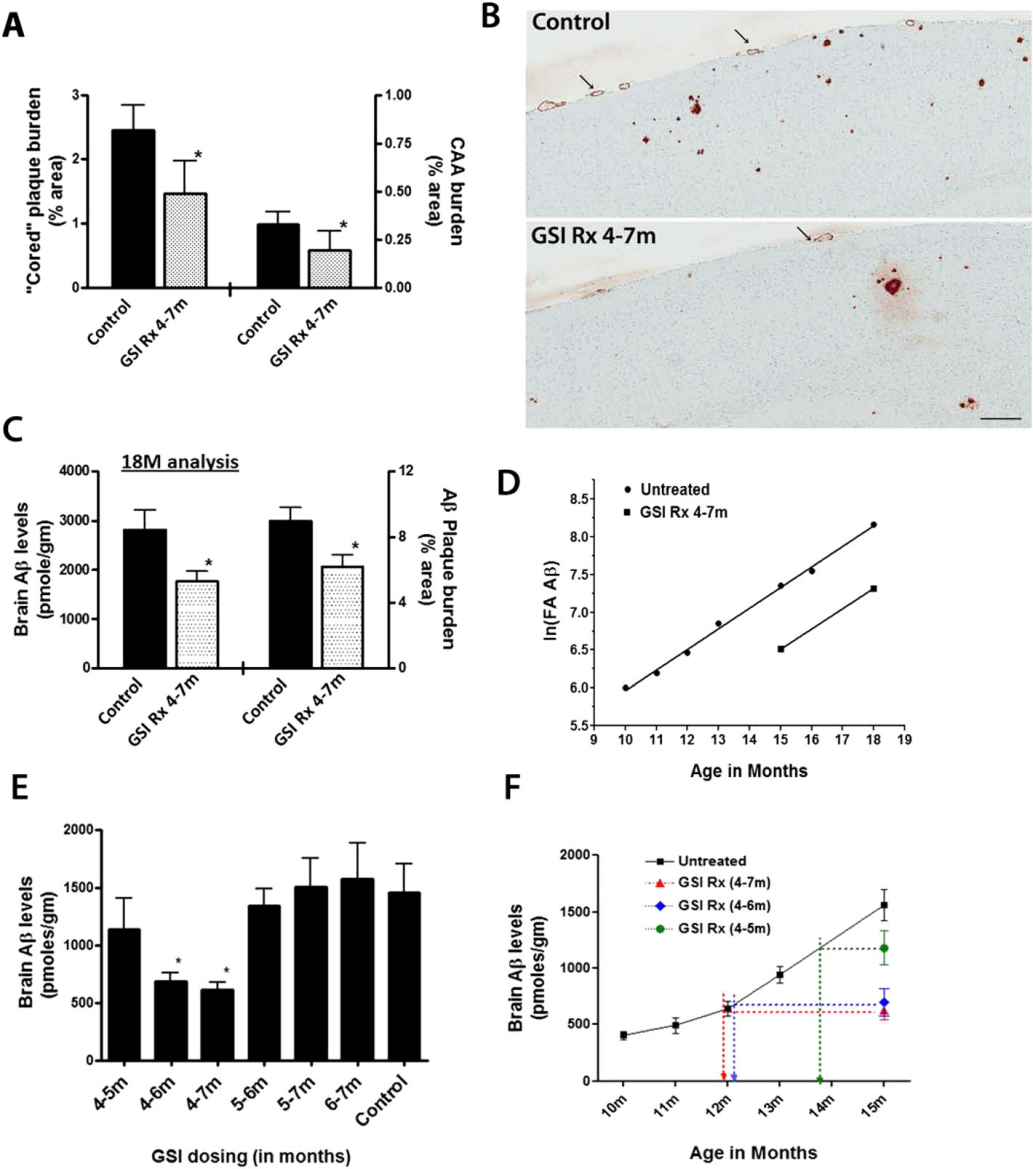
**Effects of LY window therapy on amyloid deposition. A**. Quantitative burden analysis of cored amyloid deposits and CAA in the 4-7M LY treated group compared to controls. (*p < 0.05, Student’s *t* test). **B**. Representative brain sections stained with mAb 13.1.1 showing cored plaque and CAA immune-reactivity at 15M in the brains of Control and 4-7M LY treated Tg2576 mice. Arrows indicate CAA in meningeal vessels. Scale bar = 80 μm. **C**. Biochemical analyses of FA solubilized Aβ levels and plaque burden analysis from Tg2576 mice transiently dosed with LY (2.5mpk/day) from 4-7M, and aged to 18M. (*p < 0.05, Student’s *t* test). Tg2576 mice (*n* = 5-6/group) were used, data represented is from one independent experiment. **D**. Natural logarithm transformed Aβ values of untreated Tg2576 mice (at indicated ages) and Aβ levels from 4-7M LY treatment measured at 15M and 18M of age. **E**. Biochemical analyses of FA solubilized Aβ levels of Tg2576 mice transiently dosed with LY (2.5mpk/day) at 1, 2, and 3M intervals and then aged till 15M. (*p < 0.05, ANOVA). Tg2576 mice (*n* = 5-8/group) were used, data represented is from one independent experiment. **F**. Graph representing Aβ values of untreated Tg2576 mice (from indicated ages) and Aβ levels from 4-5M, 4-6M and 4-7M LY transient treatment measured at 15M. FA solubilized brain Aβ42 + Aβ40 levels measured by ELISA are shown.

For these experiments, we transiently dosed Tg2576 mice with LY from 4-7M, 7-10M or 12-15M and aged treated mice to 15M (see Figure 1B for experimental design). To confirm efficacy of LY in all groups, we measured plasma Aβ levels immediately after the last day of treatment in sentinel mice and showed a similar reduction in plasma Aβ levels in all cohorts (Table 2). Following sacrifice at 15M, levels of Aβ in the brain were examined. The 4-7M LY treatment significantly reduced Aβ deposition; plaque burden in the frontal cortex and hippocampus was decreased by ~68% (Figure 1C) and FA-solubilized brain Aβ levels were reduced by ~60% (Figure 1D). The 7-10M LY treatment non-significantly decreased Aβ plaque burden by ~19% compared to controls (Figure 1C), whereas FA-solubilized Aβ levels were reduced by ~34% (Figure 1D). The 12-15M GSI treatment group had no significant effect on either plaque burden or FA-solubilized Aβ levels (Figure 1C, D). We further evaluated effect of the 4-7M LY treatment on cored amyloid plaques and cerebral amyloid angiopathy (CAA). Compared to untreated control mice, there were significant reductions both in cored plaques in the frontal cortex and hippocampus (~47% reduction) and CAA in the leptomeninges (~41% reduction, Figure 2A, B). To further determine whether the magnitude of the effect diminished with further aging, we performed an additional experiment, where we aged 4-7M LY treated mice to 18M, and again we observed significantly reduced Aβ plaque burden and FA-solubilized Aβ levels (Figure 2C), demonstrating that the suppression of deposition was maintained even up 11 months after treatment was halted.

**Table 2:** Plasma Aβ40 levels following transient GSI treatment in Tg2576 mice

**Treatment Time**	**Treatment Groups**	**Plasma Aβ40 (pM)**
**4-7 Month**	Ly-411,575	341.3 ± 20.1 ***(81%)***
	Control	1742.6 ± 103.5
**7-10 Month**	Ly-411,575	327.5 ± 18.5 ***(79%)***
	Control	1520.7 ± 100.8
**12-15 Month**	Ly-411,575	427.3 ± 31.4 ***(70%)***
	Control	1388.4 ± 223.2

Tg2576 mice were transiently dosed with GSI LY-411,575(2.5 mg/kg/day) at indicated time points. After GSI treatment was discontinued (on last day of treatment), plasma from groups of mice (*n* =3/group) was collected and plasma Aβ40 levels were measured by ELISA.

-Numbers in parenthesis represent percent reduction in Aβ levels.

Because Aβ deposition in mice follows an exponential course, we sought to determine how the observed reductions in Aβ accumulation correlate with Aβ accumulation to untreated mice. We compared Aβ levels in the brains of untreated Tg2576 mice from various ages ranging from 10–18 months and plotted the natural logarithm transformed value of these against Aβ levels from the 4-7M LY treated mice, aged to 15M and 18M (Figure 2D). To minimize variance, we assayed all of the samples in a single set of ELISA studies. This analysis showed that the 4 -7M GSI treatment time frame “shifts” amyloid depositing by approximately 3 months, essentially delaying Aβ accumulation by a time roughly equivalent to the treatment window.

We next attempted to determine whether this 4-7M narrow therapeutic “window” can be further refined. To do this, we transiently dosed Tg2576 mice with LY for 1, 2 or 3M intervals between the 4-7M age time frame and again aged the mice to 15M. Aβ levels were again significantly reduced in this second 4-7M LY cohort (~59% reduction) at 15M of age compared to controls (Figure 2E). Similarly, i) the 4-6M LY cohort showed a significant reduction in Aβ levels (~51% reduction) and; ii) the 4-5M LY group showed a modest but non-significant reduction in Aβ levels (~ 24% reduction) (Figure 2E). However, in the remaining 5-6M, 6-7M or 5-7M LY cohorts tested, none showed any significant reductions in Aβ levels at 15M compared to controls (Figure 2E). Since we show that the 4-7M LY “shifts” amyloid deposition equivalently to the duration of treatment, we wondered whether we will see a similar shift in amyloid deposition in the shorter treatment intervals examined that showed efficacy, e.g., in the 4-5M and 4-6M cohorts. Again, we compared Aβ levels in the brains of untreated Tg2576 mice from various ages ranging from 10–15 months against Aβ levels from the 4-5M, 4-6M, and 4-7M LY treated cohorts aged to 15M (Figure 2F). This analysis seems to show that the 4-5M GSI treatment “shifts” amyloid depositing by approximately 1 month, however, both the 4-6M and 4-7M treatment intervals essentially show a similar shift in Aβ accumulation, namely by roughly 3 months (Figure 2F). These data would suggest, at least within the 4-7M time frame that we have studied, that the 4-6M age interval may play a critical role in the early seeding phases of amyloid deposition. Additional studies, using either earlier treatment times (e.g. starting at 3M of age) or longer treatment intervals may be necessary to fully appreciate the usefulness of this early “therapeutic window” to achieve the best possible outcome on subsequent amyloid deposition.

### Transient GSI does not permanently alter APP levels or processing

**Figure 3:**
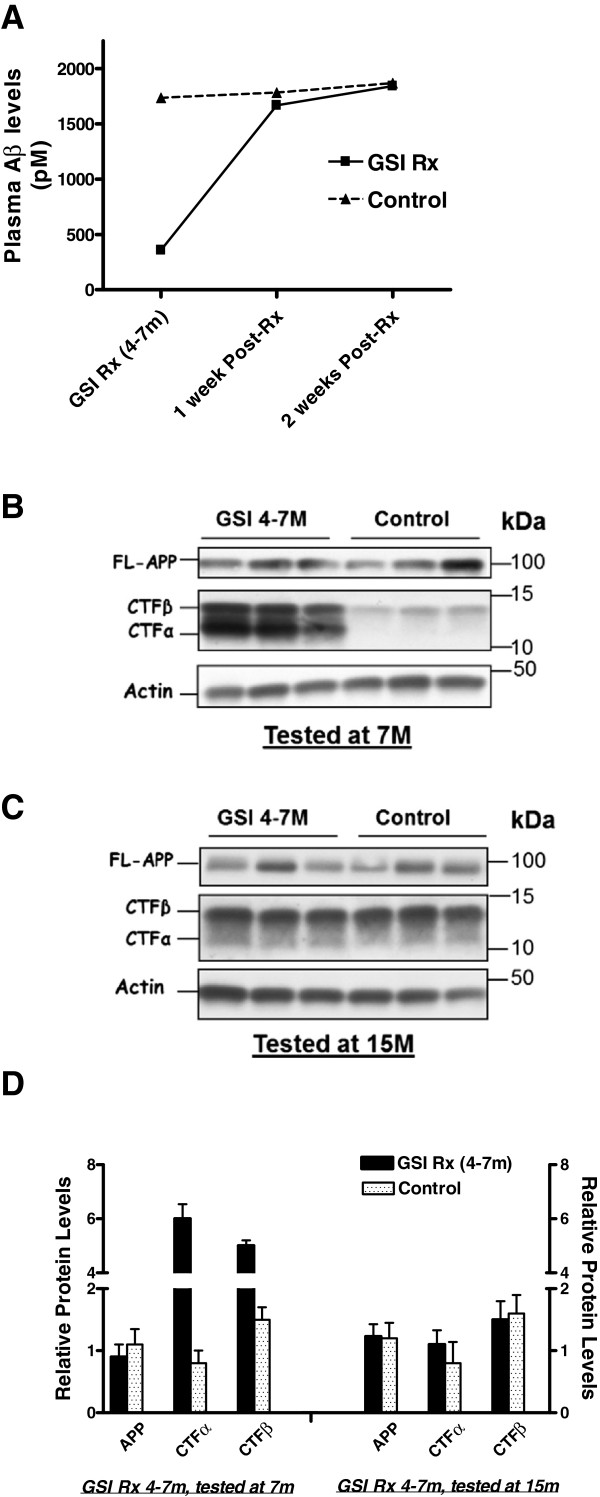
**Long term LY treatment does not permanently affect APP levels or processing. A**. Plasma Aβ40 levels by ELISA after the end of 4–7M LY treatment, (7M), 1 week after termination of treatment, and 2 weeks after termination of treatment. **B**. Representative anti-APP CT20 immunoblot shows APP levels and accumulation of CTFs after 4-7M LY treatment (tested at 7M). **C**. Representative anti-APP CT20 immunoblot showing full length APP levels and CTFs after transient 4-7M LY treatment (tested at 15M). **D**. Quantitative intensity analysis of anti-CT20 immuno-reactive full length APP and APP CTF protein levels normalized to actin.

**Figure 4:**
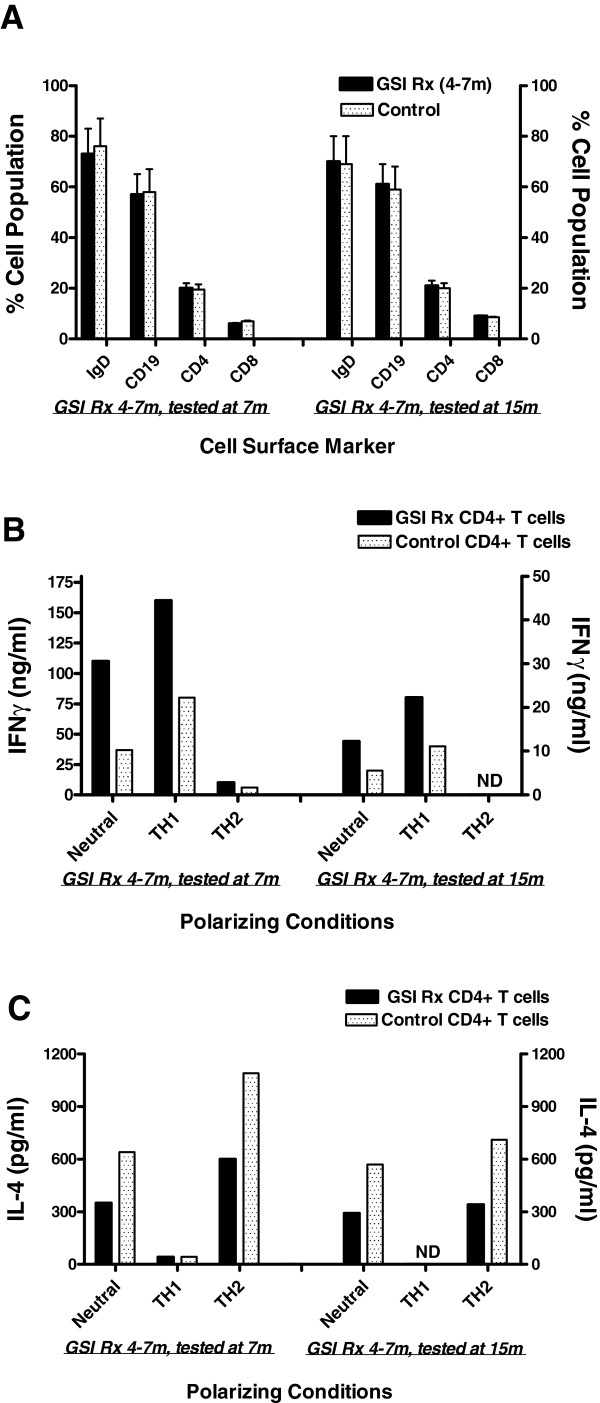
**Effect of LY treatment on peripheral immune cells.A**. Distribution of splenic B cell (IgD, CD19) and T cells (CD4 and CD8) from control and Tg2576 mice treated with LY (4-7M). Percent positive cells are shown (*n* = 4 mice/group). **B and C**. IFN production (**B**) and IL-4 production (**C**) by CD4+ T cells from LY (4-7M) treated Tg2576 mice and untreated control mice were measured by ELISA. Equal numbers of purified, splenic CD4+ T cells (pooled from n =4 mice/group) were used. ND = not determined.

To determine if the 4-7M LY treatment had long-lasting effects on Aβ production or APP levels and processing, we conducted a number of studies. We measured plasma Aβ levels at termination of GSI treatment and then determined how long before steady state levels of Aβ were normalized. Plasma Aβ levels were significantly lower immediately after treatment, but returned to control levels between with 1–2 weeks after treatment was halted (Figure 3A). We also measured levels of full-length APP and APP C-terminal fragments (CTFs) in the brain at the end of the 4-7M treatment and at 15M. Immediately following discontinuation of treatment, APP levels were unchanged but APP CTFs were increased (Figure 3B,D). However, at 15 months, there were no significant differences in full length APP or CTFs (Figure 3C,D). Finally, as GSI treatment has been shown to affect peripheral lymphocytes [13], we performed FACS analysis and quantified both B and T cell numbers in the spleen after the 4–7M LY transient treatment and at 15M. B and T cell numbers were not affected (Figure 4A). However, when we analyzed Th1 and Th2 cytokine profiles, splenic CD4+ T cells from the 4-7M LY treated cohort showed a bias towards Th1 polarization with increased IFN-γ secretion (a canonical Th1 cytokine) and concurrent decreases in IL-4 levels (a signature Th2 cytokine), which persisted even after the treatment was halted for 8 months (Figure 4B, C,). The significance of this long-lasting effect on peripheral CD4 + T cell immune responses is not clear, but certainly warrants further investigation.

## Conclusion

Our current data show that transiently lowering Aβ production in Tg2576 mice during the pre-deposition “seeding” phase has a major impact on subsequent Aβ accumulation with the effect persisting for at least 11M. Later treatment windows showed decreasing efficacy of treatment. These current data have major implications for trial design targeting Aβ production in humans. Indeed, they would suggest that efficacy in terms of plaque reduction is likely to be maximal during seeding phase, and rapidly decline if treatment is initiated during the exponential phase of deposition. A recent report [14] using passive immunotherapy provided more limited evidence that a similar effect may be observed with anti-Aβ passive immunotherapy initiated during the early deposition phase persisting for 3 months after discontinuation of treatment. Given the plethora of anti-Aβ therapies currently in development, it will be essential to determine for each modality whether efficacy is both maintained after discontinuation and similarly influenced by timing of the treatment.

The near equivalence of the delay in subsequent deposition to the length of the treatment window during a pre-deposition phase suggests that the GSI treatment from 4-7M alters or reverses seeding of Aβ. Indeed, there are no plaques forming during this period of time. Future studies will be necessary to determine the exact mechanisms by which lowering Aβ in this window alters subsequent deposition. As even *in vitro* detection of Aβ nucleation relies on indirect assays that have limited sensitivities, these studies will likely require a better understanding of nucleation and better assays to detect it. Developing such assays will be critical as direct translation of these studies would be facilitated by defining the equivalent window in humans. Regardless of mechanism, these preclinical window therapy studies have major implications regarding the potential of prophylactic therapy targeting Aβ production in humans. Not only do these data suggest that prophylactic therapy in the appropriate window will be more efficacious then initiating therapy in individuals with any preexisting Aβ pathology, but they suggest that such therapy, even if discontinued might delay subsequent onset AD by a time roughly equivalent to the time of treatment.

## Methods

### Tg2576 mice

APP Tg2576 mice were generated, maintained and genotyped as described previously [7]. All animal husbandry procedures performed were approved by the Mayo Clinic Institutional Animal Care and Use Committee in accordance with National Institutes of Health guidelines. All animals were housed three to five to a cage and maintained on *ad libitum* food and water with a 12 h light/dark cycle.

### LY-411, 575

LY-411, 575 (LY) was synthesized at the Mayo Clinic Chemical Core as previously described [15]. LY at a concentration of 16.75 mg per kg of diet was homogenously incorporated into Harlan Teklad 7012 kibble chow by Research Diets, Inc, New Jersey). Based upon dietary consumption at this age, these diets were designed to deliver 2.5 mg of LY on average per day. Tg2575 mice (n = 7-15/group/experiment) were transiently treated with LY chow at 1, 2 or 3 months interval (between 4–7 months of age), from 7–10 months of age and from 12–15 months of age. Untreated control mice from each age group were as aged and used for analysis. Groups of mice (n =3) were sacrificed immediately after the end of each treatment time-point to access efficacy of LY inhibition. After treatment was halted, mice were then aged till 15 months or 18 months of age and then sacrificed for Aβ analysis. Chow consumption, general health and body weight were monitored on a weekly basis in all treatment groups.

### Biochemical Aβ ELISA assay

Snap-frozen hemi-forebrains from Tg2576 mice were two-step sequentially extracted in 2% SDS buffer followed by 70% formic acid (FA) as described previously [16]. Aβ levels from brain lysates or plasma were the measured using sandwich ELISA techniques as described previously [16] with end specific mAbs 2.1.3 (human Aβx-42 specific, Mayo) and mAb 13.1.1 (human Aβx-40 specific, Mayo) for capture and HRP-conjugated mAb Ab5 (human Aβ1–16 specific, Mayo) for detection.

### Immunohistological analysis

Sagittal sections of paraffin embedded sections were used for analysis. Immunohistochemical staining was done using pan-Aβ antibody (mAb 33.1.1; Mayo), mAb 13.1.1 (human Aβx-40 specific, binds cored plaques and CAA; Mayo) as previously described [17]. Immunohistochemically stained sections were captured using the Scanscope XT image scanner (Aperio, Vista, CA, USA) and analyzed using the ImageScope program. Aβ plaque burden was then calculated using the Positive Pixel Count program (Aperio). At least 5 sections/brain 30 μm apart, were used and averaged by a blinded observer to calculate plaque burden.

### Western blotting

2% SDS solubilized brain lysate samples were separated on Bis-Tris 12% XT gels (Bio-Rad, Hercules, CA, USA) and probed with the antibody CT20 (anti-APP C-terminal 20 amino acid; 1:1000; P.D) and anti-actin (1:1000; Sigma, St. Louis, MO, USA). Band intensity was quantified using ImageJ software (National Institutes of Health, Bethesda, MD, USA).

### Cell isolation, *in vitro* polarization and Immuno-phenotyping

Single cell suspensions were prepared aseptically from spleens from control or GSI -treated mice. CD4+ T cells were positively selected using the BD-IMAG system and CD4 + DM particles (BD Biosciences, San Diego, CA), according to the manufacturer’s directions. Twelve-well tissue culture plates were precoated with anti-hamster Ig (Sigma), then with antibodies specific for CD3ε and CD28. Equal numbers of cells (3x10^6^) were added to each well. For TH1 polarizing conditions, anti-IL4 (10μg/ml) plus IL-12 (1 ng/ml) was added to culture wells; for TH2 polarizing conditions, anti-IFNγ (10μg/ml) plus IL-4 (1 ng/ml) was added to culture wells. No additional reagents were added to cells cultured under neutral conditions. After 24 hours, all wells were pulsed with IL-2 (10 ng/ml). After 96 hours, supernatants were collected and frozen at -80C until analyzed by ELISA for cytokine secretion. IFNγ and IL-4 cytokine secretion was measured using ELISA kits per manufactures instructions (BD Pharmingen). For cell surface staining, approximately 1x10^6^ cells (each antibody) were stained with the following antibodies individually: IgD-FITC, biotinylated anti-CD19 plus strep-PE, CD4-FITC, CD8-PE (BD Pharmingen). Data were analyzed using an LSRII flow cytometer (BD Biosciences) and FACSDiva software for acquisition and analysis.

### Statistics

All statistics were done using Graphpad Prism (Version 5.0). Comparisons of multiple groups were done by one-way ANOVA followed by Tukey's post hoc testing. Comparisons between two groups were done by Student's *t* test with Welch's correction for unequal variances where appropriate.

## Competing interests

The authors have no competing interests.

## Authors’ contributions

PD and TEG designed, analyzed and interpreted all aspects of the study and co-wrote the manuscript. CV performed all animal dosing studies, sacking and harvesting of tissues and ELISA analysis. PC performed western blotting techniques, tissue staining and quantification of plaque burdens. KF assisted with conceptualization of the study and edited the manuscript. TK performed *in vitro* cell culture analysis of LY-411, 575. GM and AF synthesized LY-411, 575 used in this study. LM and BAO performed immune cell isolation, *in vitro* polarization and immuno-phenotyping studies. All authors read and approved the final manuscript.

## Supplementary Material

Additional file 1 **Figure S1A **Wild type APP overexpressing CHO cells were treated with indicated concentrations of LY-411,575 overnight. Conditioned media were then assayed for secreted Aβ40 by sandwich ELISA. **Figure S1B.** Acute LY-411,575 treatment reduces Aβ40 levels in brains of mice. Tg2576 mice (3 month old) were injected with LY-411,575 (5 mg/kg) intraperitoneally. Mice were sacrificed 6 hrs later and brain (2% SDS solubilized) Aβ40 levels were measured ELISA. (*n*=3 mice/group) **Figure S1C, D.** Tg2576 mice were dosed orally (suspended in Kool-Aid) with LY-411,575 (10 mg/kg) for indicated times and brain (2% SDS solubilized) Aβ40 levels **(C)** and plasma Aβ40 levels **(D)** were measured by ELISA (*n*=3 mice/group). Click here for file
