# Supplementary material for: Transient pharmacologic lowering of Aβ production prior to deposition results in sustained reduction of amyloid plaque pathology
Source: Mol Neurodegener. 2012 Aug 14;7:39. doi: 10.1186/1750-1326-7-39 (PMC3477045; doi:10.1186/1750-1326-7-39)
Supplement: Additional file 1 — Figure S1A Wild type APP overexpressing CHO cells were treated with indicated concentrations of LY-411,575 overnight. Conditioned media were then assayed for secreted Aβ40 by sandwich ELISA. Figure S1B. Acute LY-411,575 treatment reduces Aβ40 levels in brains of mice. Tg2576 mice (3 month old) were injected with LY-411,575 (5 mg/kg) intraperitoneally. Mice were sacrificed 6 hrs later and brain (2% SDS solubilized) Aβ40 levels were measured ELISA. (n=3 mice/group) Figure S1C, D. Tg2576 mice were dosed orally (suspended in Kool-Aid) with LY-411,575 (10 mg/kg) for indicated times and brain (2% SDS solubilized) Aβ40 levels (C) and plasma Aβ40 levels (D) were measured by ELISA (n=3 mice/group). [file 1750-1326-7-39-S1.pdf]

Supplementary Figure 1

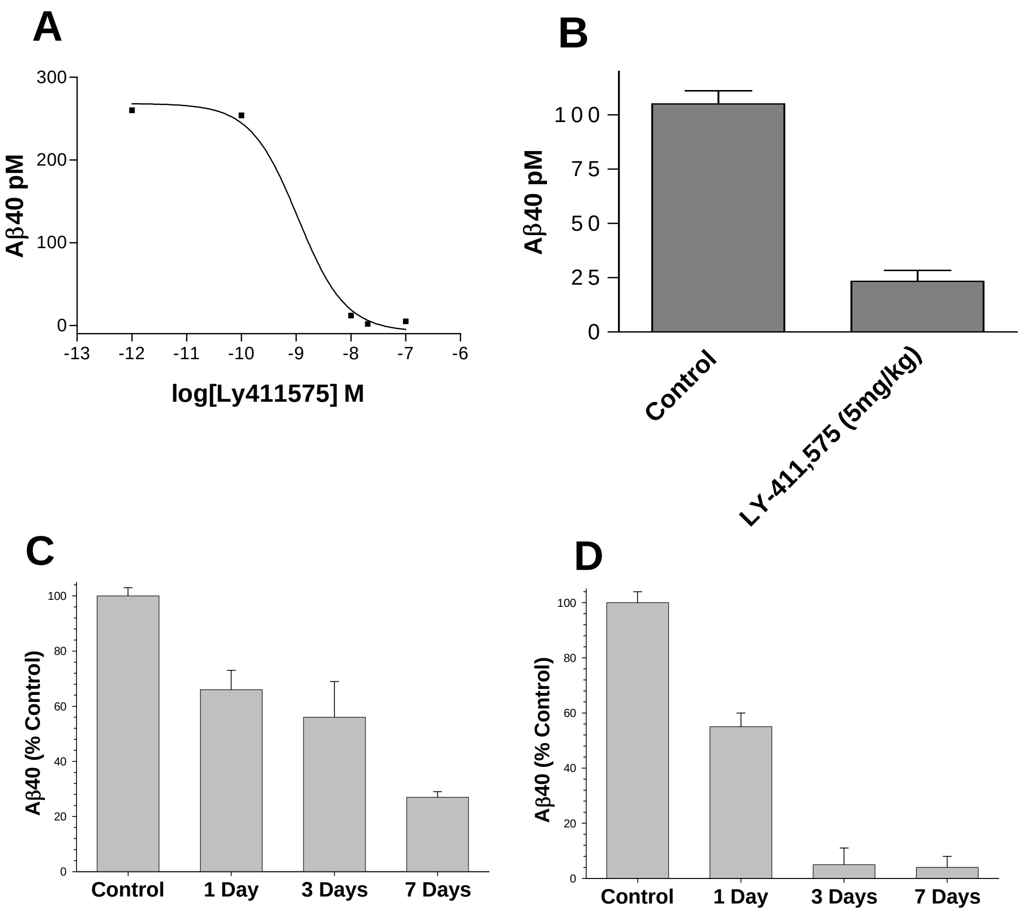

Supplementary Figure 1 Legend.

A. Wild type APP overexpressing CHO cells were treated with indicated concentrations of LY-411,575 overnight. Conditioned media were then assayed for secreted Aβ40 by sandwich ELISA.

B. Acute LY-411,575 treatment reduces Aβ40 levels in brains of mice. Tg2576 mice (3 month old) were injected with LY-411,575 (5 mg/kg) intraperitoneally. Mice were sacrificed 6 hrs later and brain (2% SDS solubilized) Aβ40 levels were measured ELISA. (*n*=3 mice/group)

C, D. Tg2576 mice were dosed orally (suspended in Kool-Aid) with LY-411,575 (10 mg/kg) for indicated times and brain (2% SDS solubilized) Aβ40 levels **(C)** and plasma Aβ40 levels **(D)** were measured by ELISA (*n*=3 mice/group).
